# The Ward’s Island “Difficulty” in New York

**Published:** 1890-04

**Authors:** 


### THE WARD'S ISLAND "DIFFICULTY" IN NEW YORK.

Our readers will recollect the references made to this affair in the January number, pages 5 and 32. This "difficulty" has now assumed a new phase.

The New York County Homœopathic Medical Society, at a session held February 13th, preferred a set of "charges" against Dr. Egbert Guernsey, President of the Hospital Board, and Dr. E. Guernsey Rankin. These charges were briefly stated by the *New York Herald* of February 15th, thus:

"Charge No. 1 says that Dr. Guernsey has spoken sneeringly of Dr. Kellogg, who is a professor in the Homœopathic College, saying that he owed his position not to any merit he possessed, but to his qualifications as an astute wire-puller. Charge No. 2 cites the fact that the doctor has on various occasions, and with malice aforethought, said that the principles of the society were based, not upon truth, but upon misrepresentation and falsehood. Charge No. 3 avers that Dr. Guernsey has spoken of Dr. Allen with contempt in saying that he had given utterance to a falsehood. Dr. Guernsey is further charged with, from time to time, ridiculing the doctrine of Homœopathy in the medical journals of which he is editor, and with giving official utterances to thoughts not in keeping with the theory of *similia similibus curantur*. All of this, of course, is held to be contrary to the spirit of a true homœopath, and consequently worthy of censure. The complaint says of Dr. Rankin that he charged the society with having fallen into disrepute and being guilty of unprofessional proceedings. It is further alleged that he misrepresented the New York County Homœopathic Medical Society in an article he recently wrote for the press and has charged openly that the society was guilty of dishonest practices."

Upon these charges Drs. Guernsey and Rankin were formally summoned to stand trial before the official board of the society at a special meeting. This meeting was held on Friday evening, February 21st, in the rooms of the society. When the meeting was convened Drs. Guernsey and Rankin appeared loaded down with bundles of manuscript and accompanied by their lawyer. The defense was made by the lawyer for Dr. Guernsey. The defense denied that the society had any right to expel the doctors.

"On the supposition that a man can only be elected a member of the County Homœopathic Society by a three-fourths vote of the society, and there being no provision in the Constitution for expelling a member save one, which relates to expulsion for the non-payment of dues, he asked by virtue of what law he was to be removed from the organization."

We quote from the *New York Herald* of Feb. 22d:

"'As to the statement that I and my friends contend simply for honesty and consistency of profession and practice, it simply brings up the old question of the meaning of the word "homœopath." If a difference of opinion as to the proper definition of the word, if a difference of opinion as to the best policy to pursue for the highest interests of medical science is to render one liable to censure and expulsion by a society which was itself organized as a protest and a revolt against high-handed bigotry, dogmatism, and ostracism, I should be glad to have the world know it. Whether it is not creditable to the society or to myself to retain my membership in the society "because I believe in maintaining all our organizations as a school and would not weaken or abandon one of them" I leave to the society without comment. Further, of what section of the medical code is this a violation? What provision therein gives this body power to discipline me?

"'The second charge,' said Dr. Guernsey, 'is based on so palpable a mistake of the types as to seriously call in question, if not the good faith of the complainants, certainly their intelligence. No one, it would seem, competent to conduct the ordinary affairs of life, unless blinded by zealous rage, would have based such a charge upon such a specification.'

"The third charge relates to an editorial which he wrote for his journal, the *New York Medical Times*. In this article Dr. Allen, a professor in the Homœopathic College, was handled without gloves. The complaint against Dr. Guernsey states that in speaking of Dr. Allen he used these words, 'cowardly, atrocious and malicious lie.'

"Dr. Guernsey submitted the editorial in question with this unique explanation:

"'I deny that I have published a "wrongful and malicious attack upon Dr. Allen." I admit that I published the words charged. They were the truthful expression of the fact. I do not palliate or excuse one word of what I published. My words properly characterized Dr. Allen's motion and action. What section of the code of ethics have I violated? What jurisdiction has this society on this matter? If I have "published a wrongful and malicious attack" upon any one surely the courts of this State are open. Will this society take cognizance of such a matter as between two men? A publication made not at a meeting of the society, but openly to the world! The dignity of the society should require that outside personal controversies should be relegated to the public tribunals established by law to remedy whatever wrongs may have been suffered.'

"In reference to another article, in which Dr. Guernsey intimated that Dr. Allen owed his position to his ability as a wire-puller and not to any special fitness he had for the office, Dr. Guernsey replied that the article was four years old, and not one of the society would call him to account for. The place to settle any such grievance, according to the doctor, was in a court of law. In reference to other articles where the doctor is charged with poking fun at honest Homœopathy, a general denial was entered as to the facts cited in the charges, with this interesting explanation by the doctor:

"'Again, let us say that for differences of opinion, honestly held, there can be no words of condemnation; but the man who has not the courage of his opinion, the man who does not dare to throw aside his professional shams, who is dishonest with himself and therefore dishonest with others, who does not love truth for truth's sake, but rather for what it will yield, merits the contempt of honest folk, and has by his own acts ostracized himself from their numbers.'

"Dr. Guernsey closed his statement by stating his position. He said he was still a good homœopath. He believed that the doctrine of *similia similibus curantur* was good as far as it went, but it did not by any means cover the whole field of medicine."

Dr. Rankin followed with his own defense, which closed in this style:

"I deny the power of the society to try me. I assert that I have had no trial. I affirm that I have committed no offense against the code of medical ethics which, if the society had the power to try me, would render me subject to its discipline."

In this struggle we see two factions holding a dispute as to which one is the true homœopath. Yet those of us who are familiar with the writings of both sides can hardly believe that either one of them clearly understands and practices pure Homœopathy.

The published utterances of the various combatants do not, in our humble opinion, display this fine knowledge. Take, for example, on the one hand, the definition of a homœopathic physician, referred to in the January number of this journal, at pages 5 and 45, and the remarks of the venerable Dr. Wells in this number.

On the other hand, take the editorials and other articles in the *New York Medical Times.*

In the light of these utterances, we do not yet see how sympathy can go out from the true Hahnemannians to either side in the controversy. W. M. J.

